# Outcome of Children with Transjugular Intrahepatic Portosystemic Shunt: A Meta-Analysis of Individual Patient Data

**DOI:** 10.1007/s00270-023-03520-z

**Published:** 2023-08-02

**Authors:** Sinan Deniz, Regina Schinner, Eric J. Monroe, Simon Horslen, Ravi N. Srinivasa, Yong Lv, Daiming Fan, Guohong Han, Moinak Sen Sarma, Anshu Srivastava, Ujjal Poddar, Rajanikant Yadav, Thi Phuong Thao Hoang, Christian M. Lange, Osman Öcal, Jens Ricke, Max Seidensticker, Eberhard Lurz, Angelo Di Giorgio, Lorenzo D’Antiga, Moritz Wildgruber

**Affiliations:** 1 Department of Radiology, University Hospital, LMU Munich, Marchioninistrasse 15, 81377 Munich, Germany; 2 Department of Radiology, University of Wisconsin, Madison, WA USA; 3 Division of Gastroenterology and Hepatology, UPMC Children’s Hospital of Pittsburgh, Pittsburgh, PA USA; 4 Division of Vascular and Interventional Radiology, Department of Radiology, University of California Los Angeles, 757 Westwood Plaza, Los Angeles, CA 90095 USA; 5 Military Medical Innovation Center, State Key Laboratory of Cancer Biology, National Clinical Research Center for Digestive Diseases and Xijing Hospital of Digestive Diseases, Fourth Military Medical University, Xi’an, China; 6 State Key Laboratory of Cancer Biology, National Clinical Research Center for Digestive Diseases and Xijing Hospital of Digestive Diseases, Fourth Military Medical University, Xi’an, China; 7 Department of Liver Diseases and Digestive Interventional Radiology, Digestive Diseases Hospital, Xi’an International Medical Center Hospital, Northwest University, Xi’an, China; 8 Sanjay Gandhi Postgraduate Institute of Medical Sciences, Lucknow, 226014 India; 9 Department of Medicine II, University Hospital, LMU Munich, Munich, Germany; 10 Division for Pediatric Gastroenterology, Hepatology and Transplantation, Department for Pediatrics, Dr. Von Haunersches Kinderspital, University Hospital, LMU Munich, Munich, Germany; 11 Paediatric Hepatology, Gastroenterology and Transplantation, Hospital Papa Giovanni XXIII Bergamo, Bergamo, Italy

**Keywords:** Cirrhosis, Transjugular intrahepatic portosystemic shunt, Portal vein thrombosis

## Abstract

**Purpose:**

The purpose of the study was to investigate outcome after pediatric transjugular intrahepatic portosystemic shunt (TIPS) with respect to survival

**Material and Methods:**

After searching for studies on TIPS in children in Ovid, Medline, Embase, Scopus and Cochrane published between 2000 and 2022, individual patient data were retrieved from five retrospective cohorts. Overall survival (OS) and transplant-free survival (TFS) were calculated using Kaplan–Meier analysis and log-rank test and compared to the indication (ascites vs. variceal bleeding) as well as to the level of obstruction (pre-hepatic vs. hepatic vs. post-hepatic). Additionally, TIPS patency was analyzed.

**Results:**

*n* = 135 pediatric patients were included in the final analysis. Indication for pediatric TIPS creation was heterogeneous among the included studies. TIPS patency decreased from 6 to 24 months, subsequent pediatric liver transplantation was performed in 22/135 (16.3%) of cases. The presence of ascites was related with poorer TFS (HR 2.3, *p* = 0.023), while variceal bleeding was not associated with impaired survival. Analysis of the level of obstruction (pre-hepatic, hepatic and post-hepatic) failed to prove significantly reduced OS for post-hepatic obstruction (HR 3.2, *p* = 0.092) and TFS (HR 1.3, *p* = 0.057). There was no difference in OS and TFS according to age at time of TIPS placement.

**Conclusions:**

The presence of ascites associates with impaired survival after TIPS in children, with no differences in survival according to the age of the child. Interventional shunt procedures can be considered feasible for all ages.

**Level of Evidence:**

Level 2a.

**Graphical Abstract:**

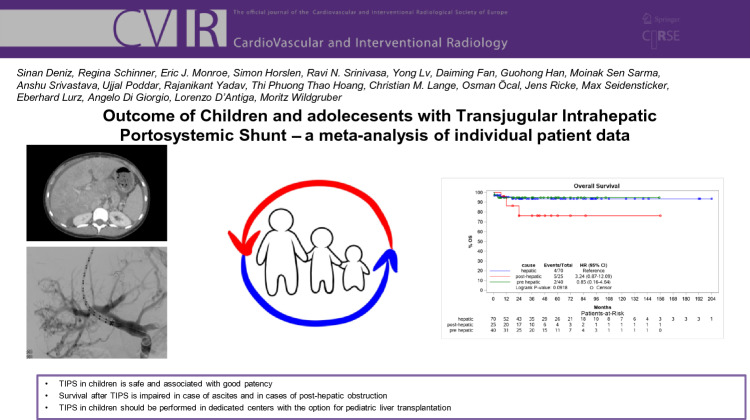

**Supplementary Information:**

The online version contains supplementary material available at 10.1007/s00270-023-03520-z.

## Introduction

Causes of portal hypertension in children differ substantially from those in adults. Extrahepatic portal vein occlusion is one of the most common reasons for portal hypertension in childhood, followed by biliary atresia [1]. While the latter may require liver transplantation in the early years of life due to liver failure, portal vein occlusion more commonly causes complications during childhood with the risk of life-threatening variceal bleeding, but synthetic function frequently being preserved. These patients frequently do not become eligible for liver transplantation and require different therapies [2].

Current management of childhood portal hypertension includes medical, endoscopic surgical and interventional radiology techniques, all of which can serve as a bridge to liver transplantation. Surgical shunt procedures such as Meso-Rex bypass, mesocaval shunt and splenorenal shunt are established techniques [3], but long-term patency may be limited [4]. Meso-Rex bypass is always to be preferred in children with PVT, but it may not be possible in cases of portal vein thrombosis extending to the intrahepatic portal vein segment or in emergency situations [5, 6]. Similarly, surgical shunt procedures may not be eligible in case of cirrhosis or congenital hepatic fibrosis.

Transjugular intrahepatic portosystemic shunt (TIPS) creation is a good alternative to surgical shunt procedures being less invasive and, at least in the adult population, associated with higher patency rates [7].

Within the past decade, a variety of retrospective single-center cohorts have been published investigating the safety, effectiveness and clinical outcome of TIPS in children. Prospective clinical trials are not available and owing to the rarity of the disease are unlikely to become available in the near future. We aimed to analyze the outcome of TIPS creation in children with a focus on survival and with respect to different levels of portal hypertension and resulting indications for TIPS.

## Material and Methods

### Search Methods, Study Selection and Data Collection

Two authors (SD and MW) identified potentially eligible studies by searching electronic literature databases on Ovid, Medline, Embase, Scopus and Cochrane from 2000 until 2022. Further eligible studies were collected by screening the bibliographies of retrieved manuscripts. Disagreements on study inclusion were solved by discussion between the two reviewers. Pre-defined eligibility criteria for studies were as follows: (1) inclusion of ≥ 10 patients; (2) successful TIPS creation in children and adolescents aged 1–20 years and (3) report of survival and potential subsequent liver transplantation. We invited all corresponding, first and last authors of publications from eligible cohorts to share anonymized data at the individual patient level. Requesting data at the individual patient level allowed to collect missing additional data required for the meta-analysis which was not necessarily included in the original publication. Case reports and small retrospective cohorts with < 10 patients reported were excluded.

Besides demographic data, the following data at baseline were collected: presence of portal vein thrombosis (PVT) or Budd–Chiari syndrome (BCS), presence of cirrhosis, variceal bleeding, ascites, encephalopathy, genetic coagulation disorders, laboratory values (liver function tests, albumin, thrombocytes, INR, sodium and creatinine), procedural characteristics such as stent type and shunt diameter, anticoagulation/platelet inhibition post-TIPS, patency, reintervention, survival (overall and transplant free), time to transplantation and maximum follow-up. Pediatric end-stage liver disease (PELD) score was calculated accordingly. Additionally, the primary indication for TIPS was analyzed. For analyses and preparation of the manuscript, we followed the reporting guidelines of the meta-analysis of observational studies in epidemiology (MOOSE) [8].

The local ethics committee waived the need for both individual consent or specific approval of the meta-analysis due to fully anonymized data.

### Outcome Definitions

Primary outcome was defined as overall survival following TIPS. As secondary outcome measures transplant-free survival in case of pediatric liver transplantation was chosen, as well as TIPS patency. Both overall (OS) and transplant-free survival (TFS) were assessed and compared according first to primary TIPS indication (ascites or variceal bleeding) as well as according to the level of obstruction (pre-hepatic, hepatic and post-hepatic). Additionally, OS and TFS were analyzed according to the different age groups: pre-schooler (2–6 years), child (7–12 years) and youth (13–20 years). TIPS patency was assessed on a biannual basis at each center. Follow-up was defined as last presentation of the patient or time of death/liver transplantation.

### Statistical Analysis

Baseline characteristics were compared between different patient groups (primary indication, age groups, etc.) using either *t*-test/ANOVA for continuous parameters or Chi-square test/Fisher’s exact test for categorical data. Overall survival (OS) was calculated from TIPS creation until death. Likewise, transplant-free survival (TFS) was calculated until transplant, or in case of no transplant, until death. In the absence of transplant/death, OS and TFS were censored at the last available follow-up.

Kaplan–Meier analyses were performed for OS and TFS, and log-rank test was used to compare different primary TIPS indications and level of obstruction as well as age groups. Statistical analyses were performed using SAS version 9.4 for Windows (SAS Institute, Inc.). A *p* value < 0.05 was considered to indicate statistical significance.

## Results

### Studies and Data Included

**Fig. 1:**
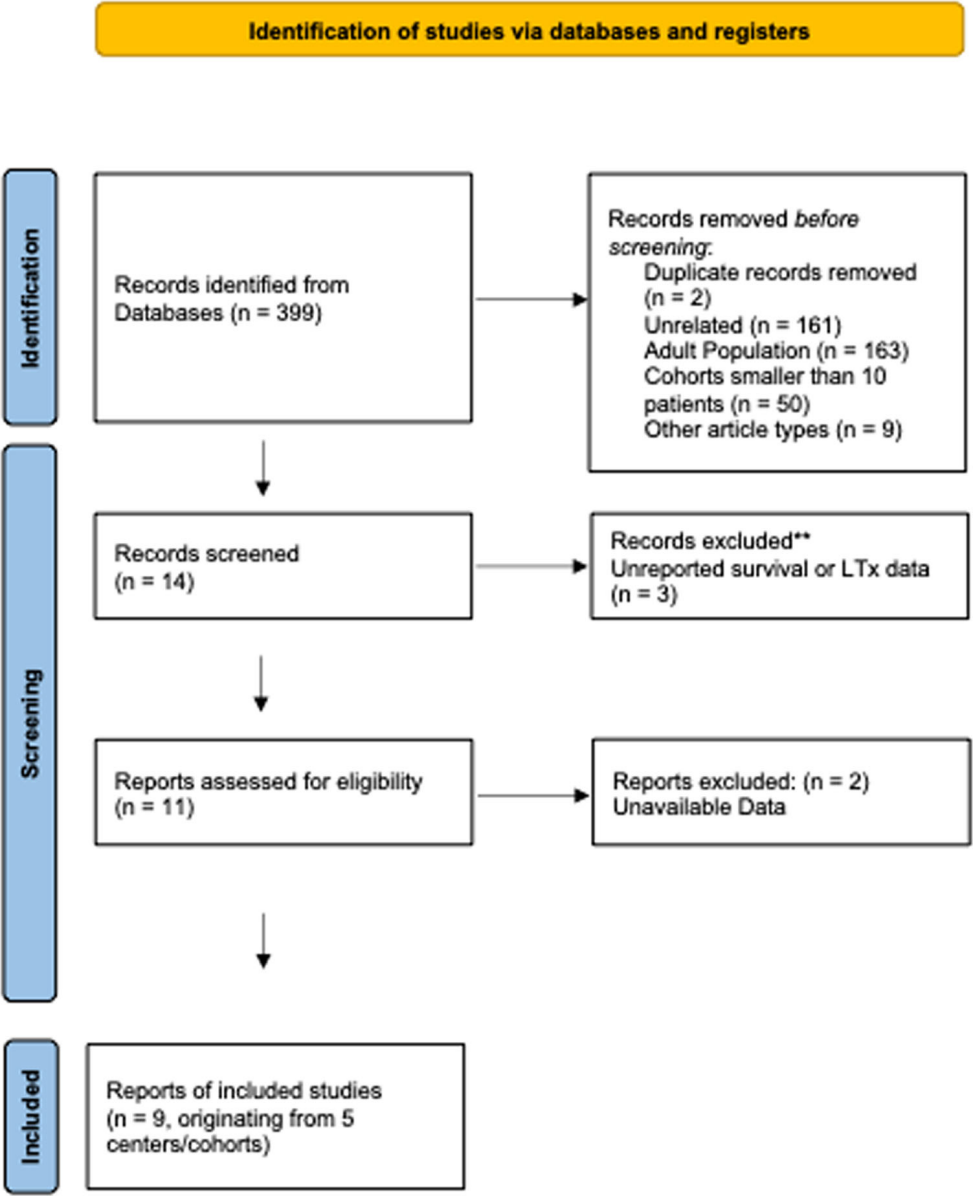
PRISMA diagram. Flowchart of the studies finally included in the meta-analysis according to the preferred reporting items for systematic review and meta-analyses (PRISMA)

Data from nine published retrospective reports were included, originating from five pediatric liver centers (Fig. 1): two cohorts from the US [9–11], one Italian cohort [12–14], one Indian cohort [15, 16] and one Chinese cohort [17]. Two patients from the Italian cohort with unsuccessful TIPS creation were not included in the subsequent analyses. A quality assessment of included studies is provided in Supplementary Table 1.

### Demographic Data

Of the 135 patients included in the meta-analysis, 71 (52.6%) were male, and 64 (47.4%) children were female. Mean age was 12 ± 4 years. Twenty-two (16.3%) patients were grouped as pre-schooler (2–6 years), 49 (36.3%) as child (7–12 years) and 64 (47.4%) as youth (13–20 years). Demographic data of the included patient cohorts, shown for the entire cohort as well as separately for each cohort included, are summarized in Supplementary Table 2. Laboratory values at the time of TIPS creation are reported in Supplementary Table 3. Mean follow-up period reported after pediatric TIPS creation was 45.5 ± 44.2 months with FU times ranging from 1 month to 20 years. Genetically proven coagulation disorders were assessed in 42/135 patients, with at least one proven coagulation disorder reported in 15 (35.7%) and a negative test in 27 (64.3%, *p* = 0.347), but also significant differences between the cohorts (*p* = 0.0223). Underlying diseases are presented in Table 1 with portal vein thrombosis and Budd–Chiari syndrome being the most frequent diseases requiring pediatric TIPS creation. Underlying diseases and subsequent indications differed significantly between the cohorts included, e.g., in two cohorts, BCS was the most frequent underlying disease, while two studies did not include TIPS creation in BCS at all (*p* < 0.0001).

**Table 1:** Disease distribution according to the level of obstruction and indication for TIPS

	(1) Portal vein thrombosis	(2) Budd–Chiari/VOD	(3) Congential hepatic fibrosis	(4) Sclerosing cholangitis	(5) Cystic fibrosis	(6) IFALD	(7) Biliary atresia	(8) Hepatoportal sclerosis	(9) Cryptogenic cirrhosis	(10) Biliary cirrhosis	(11) ARPCKD	Total
*Level*												
Pre-hepatic	40	0	0	0	0	0	0	0	0	0	0	40
Hepatic	0	0	4	6	8	2	20	2	21	2	5	70
Post-hepatic	0	25	0	0	0	0	0	0	0	0	0	25
*Variceal bleeding*												
No	6	16	2	0	1	0	2	1	0	0	1	29
Yes	34	9	2	6	7	2	18	1	21	2	4	106
*Ascites*												
No	29	4	2	6	6	2	17	1	21	2	4	94
Yes	11	21	2	0	2	0	3	1	0	0	1	41
*Total*	40	25	4	6	8	2	20	2	21	2	5	135

Underlying diseases of all patients included are summarized and distribution according to the level of obstruction (pre-hepatic, hepatic and post-hepatic), and TIPS indication (ascites and variceal bleeding) is presented.

VOD, veno-occlusive disease; IFALD, intestinal failure associated liver disease and ARPCKD, autosomal recessive polycystic kidney disease

Cirrhosis was reported in 47/135 patients (34.8%), and there were significant differences between the five cohorts (India 100%, Italy 40.7%, the USA-1 26.3%, the USA-2 28.1% and China 0% cirrhotics; *p* < 0.0001). Also, age groups (16.3% pre-schoolers vs. 36.3% children vs. 47.4% youths) differed significantly between the cohorts (*p* = 0.006).

### Indication for TIPS

TIPS creation was performed in 106/135 cases (78.5%) with variceal bleeding, in 41/135 cases (30.3%) with ascites.

### TIPS Technique

TIPS creation was performed under general anesthesia following standard operating procedures at the corresponding centers. Transjugular access via the hepatic vein was obtained in 109/135 (80.7%) of patients, in 26 (19.3%), a combined percutaneous and transjugular access was used to gain access to the portal vein. TIPS stents were sized from 5 to 10 mm, only in one case, a 14 mm sized shunt was created, with a median shunt diameter of 10 mm. Anticoagulation or platelet inhibition was reported from two of five centers using either warfarin or acetylsalicylic acid, being administered for 6 months following TIPS.

### TIPS Patency

A patent TIPS, meaning an open shunt documented at follow-up, was reported in 96/135 (71.1%) at 6 months, in 78/135 (57.8%) at 12 months and in 58/135 (43%) after 24 months follow-up. Non-patent TIPS was reported as follows: A total of four children experienced TIPS occlusion in the first 6 months, five within the first 12 months and 8/135 children had TIPS occlusion within 24 months. Detailed analysis of TIPS patency is reported in Table 2 comparing patency rates according to the level of obstruction as well as according to the indication. TIPS patency differed significantly between the different levels of obstruction (pre-hepatic, hepatic and post-hepatic) at all time points. Complete patency data are included in Supplementary Table 2, including patients lost to follow-up as well as children having undergone liver transplantation, with no patency data to calculate in those cases. Mean time period of TIPS recanalization after creation in case of occluded TIPS was 13.9 ± 16.3 months. Reported primary patency rates with respect to age of the children differed significantly only at 6 months (*p* = 0.0139) where pre-schoolers had a worse patency rate of 45.5% compared to children 83.7% and youths 70.3% though, with differences at 12 (*p* = 0.308) and 24 months (*p* = 0.258) being non-significant. As the type, dose and duration of anticoagulation were not routinely reported in the studies, a comparison between children with and without anticoagulation after TIPS was not performed.

**Table 2:** TIPS patency according to the level of obstruction and the two main indications ascites and variceal bleeding

TIPS patency	Total (*N* = 135)	Pre-hepatic (*N* = 40)	Hepatic (*N* = 70)	Post-hepatic (*N* = 25)	*p* value	Variceal bleeding (*N* = 106)	No variceal bleeding (*N* = 29)	*P* value	Ascites (*N* = 41)	No ascites (*N* = 94)	*p* value
6 months					0.0007			0.7645			0.0293
No	4 (3.0)	1 (2.5)	1 (1.4)	2 (8.0)		3 (2.8)	1 (3.4)		3 (7.3)	1 (1.1)	
Yes	96 (71.1)	32 (80.0)	41 (58.6)	23 (92.0)		74 (69.8)	22 (75.9)		32 (78.0)	64 (68.1)	
na/LTx/LTFU	35 (25.9)	7 (17.5)	28 (40.0)	0 (0.0)		29 (27.4)	6 (20.7)		6 (14.6)	29 (30.9)	
12 months					0.0485			0.9378			0.2358
No	5 (3.7)	2 (5.0)	1 (1.4)	2 (8.0)		4 (3.8)	1 (3.4)		3 (7.3)	2 (2.1)	
Yes	78 (57.8)	27 (67.5)	34 (48.6)	17 (68.0)		62 (58.5)	16 (55.2)		25 (61.0)	53 (56.4)	
na/LTx/LTFU	52 (38.5)	11 (27.5)	35 (50.0)	6 (24.0)		40 (37.7)	12 (41.4)		13 (31.7)	39 (41.5)	
24 months					0.0040			0.2408			0.0169
No	8 (5.9)	2 (5.0)	1 (1.4)	5 (20.0)		5 (4.7)	3 (10.3)		6 (14.6)	2 (2.1)	
Yes	58 (43.0)	21 (52.5)	26 (37.1)	11 (44.0)		49 (46.2)	9 (31.0)		15 (36.6)	43 (45.7)	
na/LTx/LTFU	69 (51.1)	17 (42.5)	43 (61.4)	9 (36.0)		52 (49.1)	17 (58.6)		20 (48.8)	49 (52.1)	

Patency of pediatric TIPS is given at 6, 12 and 24 months and distribution according to the level of obstruction (pre-hepatic, hepatic and post-hepatic) and TIPS indication (ascites and variceal bleeding) with corresponding *p*-values

### Survival Analysis According to TIPS Indication

**Fig. 2:**
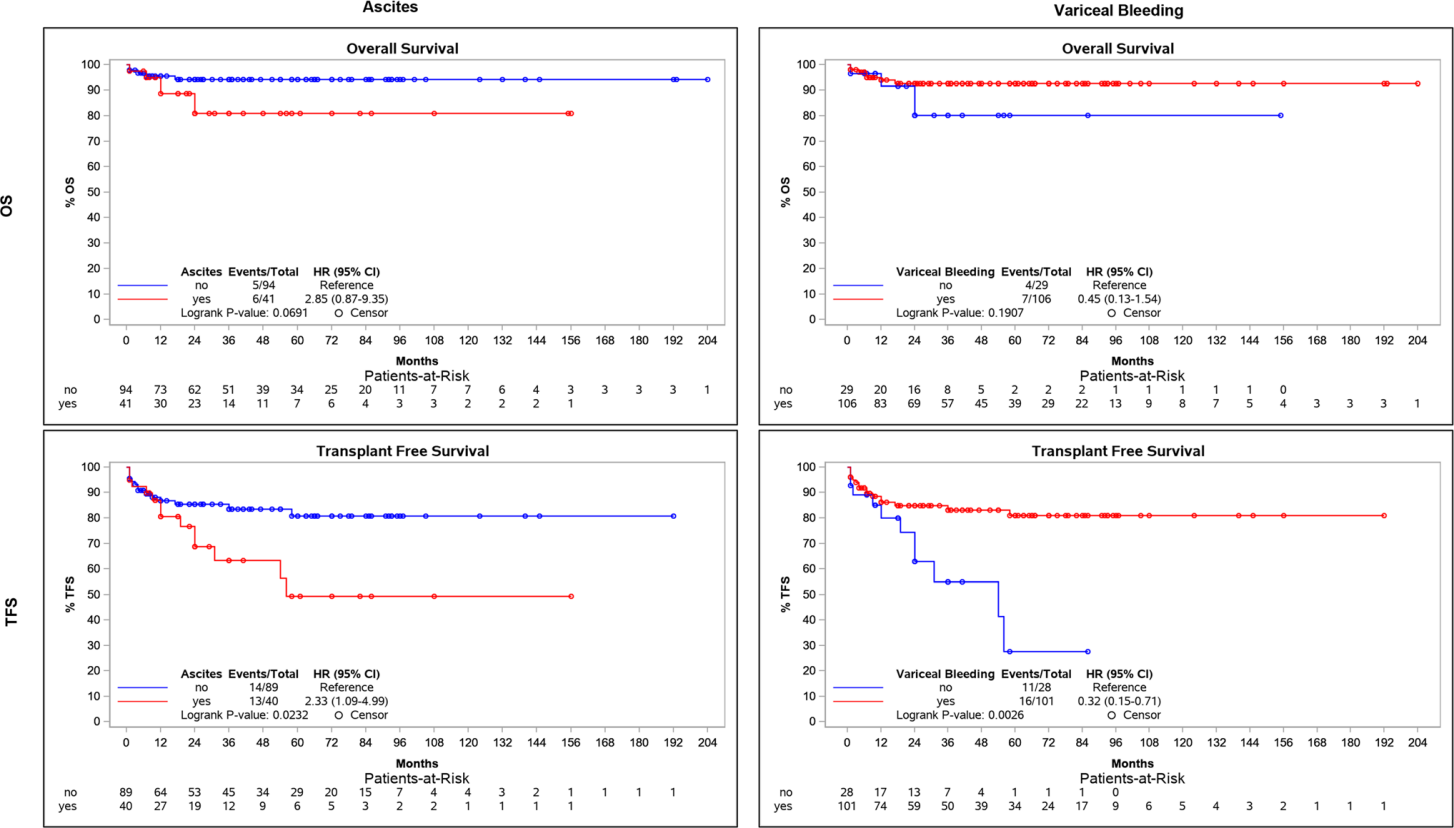
Survival analysis according to the indication for TIPS. Figure shows Kaplan–Meier plots for both overall survival (OS, first row) and transplant-free survival (TFS, second row) with respect to the primary indication for TIPS: ascites (first panel) and variceal bleeding (second panel). Hazard ratios (HR) and *p*-values for the log-rank test are included

OS and TFS were analyzed with respect to the primary indication: ascites and variceal bleeding (Fig. 2). Ascites was associated with impaired TFS (HR = 2.33, *p* = 0.023), but not OS (HR = 2.85, *p* = 0.069). While variceal bleeding was not associated with OS (HR = 0.45, *p* = 0.191), the statistical significance with respect to TFS has to be interpreted with caution due to the low number of events (HR = 0.32, *p* = 0.002).

### Survival Analysis According to the Underlying Level of Obstruction

**Fig. 3:**
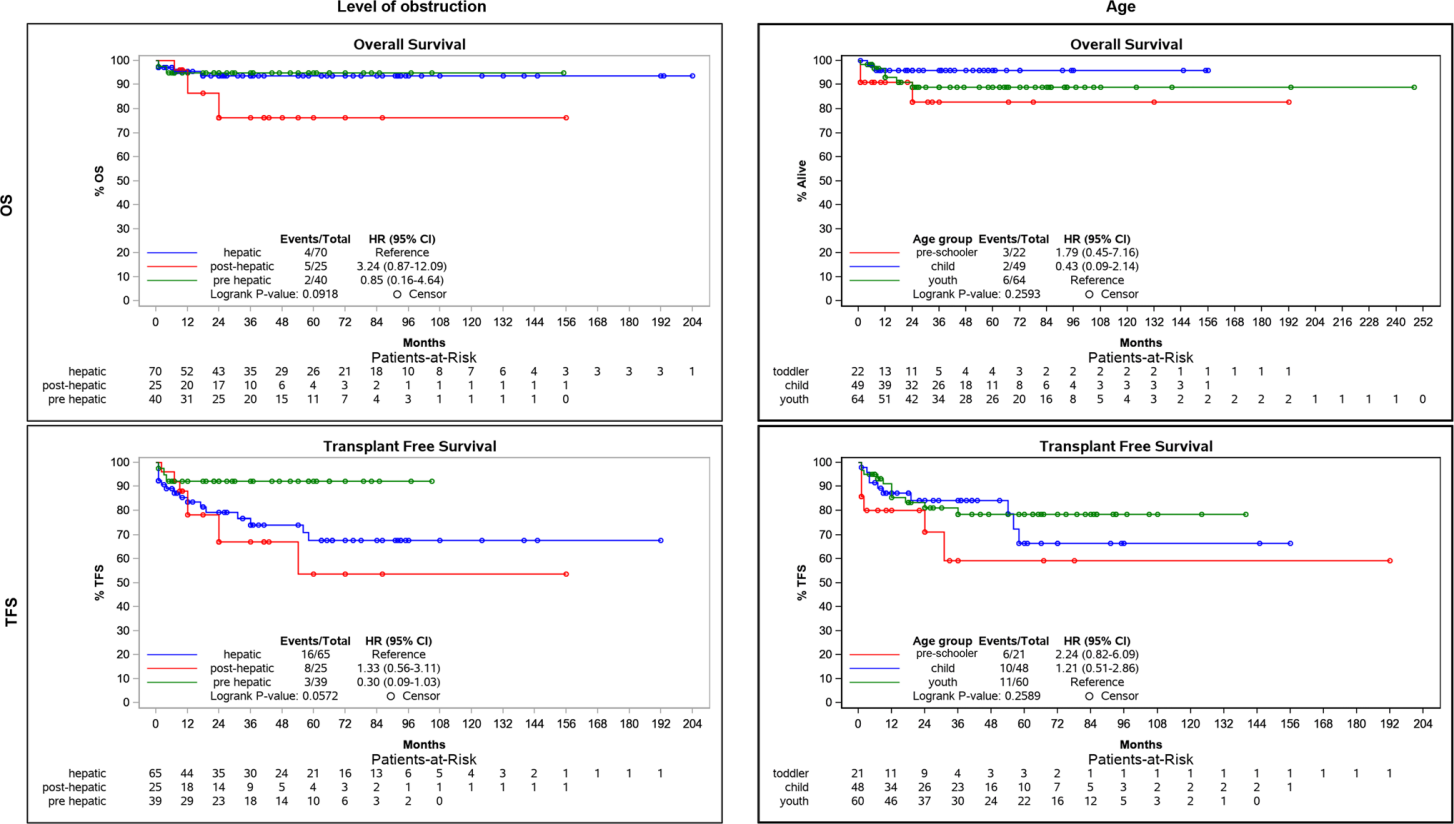
Survival analysis according to the level of obstruction and patient age. Figure shows Kaplan–Meier plots for both overall survival (OS, first row) and transplant-free survival (TFS, second row) with respect to the level of obstruction (pre-hepatic, hepatic and post-hepatic, left panels) and patient age (pre-schooler, child and youth, right panels). Hazard ratios (HR) and *p*-values for the log-rank test are included

OS and TFS were additionally analyzed with respect to the level of obstruction: pre-hepatic, hepatic and post-hepatic (Fig. 3, left panels). Post-hepatic obstruction, including the patients with BCS, did not prove significantly reduced OS (HR 3.2, *p* = 0.092) and TFS (HR 1.3, *p* = 0.057). The presence of cirrhosis was not associated with OS (HR = 0.64, *p* = 0.565) nor TFS (HR 2.0, *p* = 0.065).

#### OS Analyses According to Patient Age

OS analyses with respect to the patient age (pre-schooler, child and youth, Fig. 3 right panels) revealed no significant differences both for OS and TFS (*p* = 0.259).

#### Survival Dependence from PELD and Liver Transplantation

**Fig. 4:**
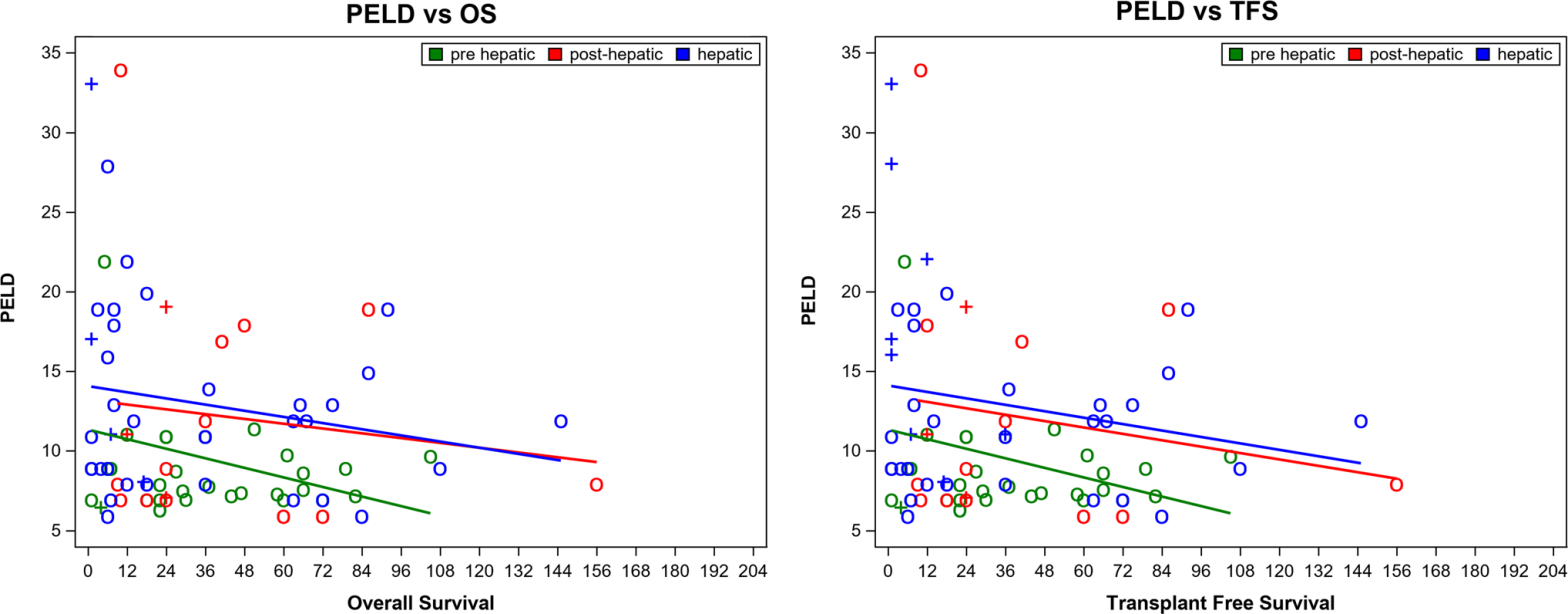
Survival analysis according to PELD score. Scatter plots show individual patients with values for OS (left panel) and TFS (right panel) according to the level of obstruction (pre-hepatic in green, hepatic in blue and post-hepatic in red). +  = transplant or death and o = censored

PELD score was negatively associated with both OS and TFS (Fig. 4), for all levels of obstruction. Pediatric liver transplantation was performed in 22/135 (16.3%) of children after TIPS. Mean period from TIPS creation to liver transplantation was 22.9 ± 26.9 months, with no difference between the three age groups (*p* = 0.226).

Mean PELD score at time of transplantation was 17.1 ± 9.4 compared to children not being transplanted (10.6 ± 4.9, *p* = 0.018). Children who died in the course after TIPS had a PELD score of 16.2 ± 10.5, while children with no death reported had a PELD of 11.6 ± 6.2 (*p* = 0.246).

## Discussion

Shunt procedures lead to a rapid decrease in portal hypertension. In adults, a poorer patency of surgical shunt procedures has led to TIPS being the primary choice to decrease portal hypertension [18]. Yet, these data cannot be extrapolated to the pediatric patient population with special procedures such a Meso-Rex shunts being available. Moreover, patency rates for both surgical and interventional shunts have not been compared directly to each other and can only be derived from retrospective cohorts [3, 9–13, 17, 19, 20]. A recent meta-analysis investigating the technical success and patency rates for TIPS in children reported technical success rates above 90% and good patency rates (pooled patency 84%) [21], yet this work did not focus on survival. Clinical outcome and survival after shunt procedures do not merely depend on shunt patency, but on the progression of the disease, which is particularly important in children having their entire lifespan still ahead. Besides focusing on survival, our meta-analysis is based on individual patient specific data, with the advantage of avoiding double publication of patients included in more than one study and allowing further analysis beyond data presented in the original studies.

This meta-analysis revealed that the presence of ascites was associated with impaired survival, whereas variceal bleeding was not. In adults, early TIPS creation in case of acute variceal bleeding improves the survival period to liver transplantation [22]. These results, however, are not contradictory to those of this meta-analysis in children. In our comparison, the group without variceal bleeding contained a substantial number of children with ascites, with particularly impaired survival. TIPS in children with variceal bleeding may still be beneficial to rescue patients in the early course following a bleeding episode, which, however, needs to be evaluated in the future studies. With respect to the level, post-hepatic obstruction showed worst survival rates, essentially representing children with BCS. TFS was additionally decreased in hepatic obstruction compared to pre-hepatic obstruction, whereas OS was similarly between both. Age of children at time of TIPS creation showed no association, neither with OS nor TFS.

With continuously improving outcomes of pediatric liver transplantation, especially of living donor transplantation [19], the perspective of children with portal hypertension is improving. With only 22/130 patients having received liver transplantation after TIPS creation in the overall cohort, it similarly becomes obvious that limited resources and availability of pediatric liver transplantation limit the outcome of children with portal hypertension. Differences in OS and TFS in this analysis have to be interpreted with caution, as obviously access to liver transplantation was different across centers. Not surprisingly, PELD scores negatively associated with survival, across the various levels of obstruction.

The reported patency rates after pediatric TIPS declined from 6 to 12 to 24 months. The rate of TIPS occlusions at 24 months being ~ 5% suggests that TIPS occlusions in children are similarly rare as in adults. Patency rates reported here, however, have to be interpreted with the knowledge that only ~ 70% of TIPS were created using stentgrafts, which are known to substantially alter TIPS patency. Patency rates differed significantly across the different centers. Additionally, substantial differences between the levels of obstruction were observed with interestingly worst patency rates observed for hepatic obstruction. Of note, patency was not assessed via standardized protocols across centers, incorporating different modalities and time points for determining patency. Shunt diameters were obviously sized independent from age, which may have additional impact on patency. Patency additionally depends on the type of shunt creation (bare stents versus covered grafts). While (partially) covered stents are the standard of care in adult patients, TIPS in children frequently requires improvised individual constructs, especially in BSC/PVT making an analysis according to the type of shunt creation complex and hard to interpret.

Anticoagulation in pediatric TIPS was variable between centers. Unfortunately, details about anticoagulation were not routinely reported, making subsequent analyses difficult to interpret. Currently, the EASL guidelines state that, even for adults, there is no agreement on whether anticoagulation after TIPS is necessary at all [23]. The decision for anticoagulation further depends on the underlying disease, with BCS or PVT certainly requiring anticoagulation [23]. A recent retrospective multicenter study showed a potential survival benefit for acetylsalicylic acid after TIPS in adults [24], although this opinion is not without detractors [25]. In children, there is no consensus, and the primary indication for TIPS may guide anticoagulation.

PVT was a relatively common etiology in this analysis, however with the age of thrombosis not known. It should be considered however that portal vein recanalization can not only be achieved via TIPS but also similarly with surgical and endovascular approaches without a portosystemic shunt [26]. Although the performance of TIPS with portal vein recanalization in PVT in adults seems to be favorable compared to recanalization without portosystemic shunt [27], these data again cannot be extrapolated to the pediatric population. The interpretation of outcomes after TIPS in PVT data was particularly challenging with respect to underlying cirrhosis. From the published original data, it was not obvious if PVT developed on the ground of underlying cirrhosis or if cirrhosis was a consequence of chronic PVT. The PVT cases with cirrhosis in this cohort might represent progressive liver disease complicated by PVT as a specific disease entity. Moreover, the etiology of PVT may vary across countries. Therapy regimens in PVT are similarly heterogeneous, including again the variable handling of pediatric liver transplantation, which is expected to impact the outcome of PVT differently in varying health-care settings.

Over 25% of children displayed some degree of liver cirrhosis. Cirrhosis is expected to increase also in the pediatric population due to multiple reasons such as increase in non-alcoholic steatohepatitis and increasing survival rates of congenital liver diseases.

The major limitation of this meta-analysis is the limited number of patients included with, moreover, a very heterogeneous spectrum of diseases included as well as varying therapeutic approaches including liver transplantation. This heterogeneity had substantial impact on the analysis, with a potential confounding of data. As stated, the reported cohorts from Europe, the US and Asia differed in terms of subsequent liver transplantation; thus, TFS data have to be interpreted carefully. Missing values from the retrospective cohorts, with, e.g., cirrhosis grading not being reported in each cohort, moreover, led to impaired statistical analyses. A higher number of censored observations compared to the moderate number of events (deaths/transplantation) made reasonable sub-analyses such as time-to-event impossible.

The applied methodology, by including original data provided by the authors, has a lot of advantages; however, as certain data were not available from the original data, some initially planned analysis such as impact on different levels on cirrhosis on outcome after TIPS could not be performed. Complications of TIPS creation such has right heart overload as well as hepatic encephalopathy were not routinely reported and certainly require further investigation. Another major drawback when interpreting the outcome after TIPS creation in children is the lack of an equivalent control group, with similar underlying disease, but either not undergoing portosystemic shunt treatment or children treated with surgical shunt procedures.

Given the rarity of the underlying disease, prospective controlled trials investigating TIPS in children are unlikely to take place in the foreseeable future; however, prospective registries investigating the course and clinical outcome in rare liver diseases are being established any may provide additional evidence [28, 29].

## Conclusion

In summary, this meta-analysis proves that TIPS can be effectively performed in children. Patency rates are comparable to TIPS in adults but may have only minor impact on survival, which is mostly determined by the underlying liver disease.

## Supplementary Information

Below is the link to the electronic supplementary material.

Supplementary file1 (DOCX 23 KB)

Supplementary file2 (XLSX 22 KB)

Supplementary file3 (XLSX 20 KB)
