# Supplementary material for: Outcome of Children with Transjugular Intrahepatic Portosystemic Shunt: A Meta-Analysis of Individual Patient Data
Source: Cardiovasc Intervent Radiol. 2023 Aug 2;46(9):1203–13. doi: 10.1007/s00270-023-03520-z (PMC10471675; doi:10.1007/s00270-023-03520-z)
Supplement: Supplementary file 1 — Supplementary file1 (DOCX 23 KB) [file 270_2023_3520_MOESM1_ESM.docx]

**Table. Quality Assessment of the included studies**

|  | Selection | Ascertainment | | Causality | | | | Reporting | Total  (max.8) |
| --- | --- | --- | --- | --- | --- | --- | --- | --- | --- |
| Author/Year^#^ | Does the patients represent the whole experience of centre? | Was the exposure adequately ascertained? | Was the outcome adequately ascertained? | Were other alternative causes ruled out? | Was there a challenge/rechallenge phenomenon? | Was there a dose-response effect? | Was follow-up long enough? | Is the cases described with sufficient details? |  |
| Di Giordio 2020 | * | * | * | * |  |  | * | * | 6 |
| Slowik 2019 | * | * | * | * |  |  | * | * | 6 |
| Vo 2012 | * | * | * | * |  |  | * | * | 6 |
| Ghannam 2018 | * | * | * | * |  |  | * | * | 6 |
| Author/Year^†^ | **Selection** | | | | **Comparability** | **Exposure** | | | Total  (max.9) |
|  | Is the Case Definition Adequate? | Representativeness of the Cases | Selection of Controls | Definition of Controls | Cohorts on the basis of design or analysis | Ascertainment of Exposure | Same method | Non-Response Rate |  |
| Lv 2015 | * | * |  |  | ** | * | * |  | 6 |
| Singh 2018 | * | * |  | * | ** | * | * |  | 7 |
| Kathuria 2014 | * | * |  |  | ** | * | * |  | 6 |
| Quality assessment of case-control studies^†^ was performed using the Newcastle-Ottawa Scale and of case series^#^ was performed using an adaptation of this scale (Murad MH et al, BMJ Evid Based Med. 2018). | | | | | | | | | |
